# Biology, physiology and gene expression of grasshopper *Oedaleus asiaticus* exposed to diet stress from plant secondary compounds

**DOI:** 10.1038/s41598-017-09277-z

**Published:** 2017-08-17

**Authors:** Xunbing Huang, Jingchuan Ma, Xinghu Qin, Xiongbing Tu, Guangchun Cao, Guangjun Wang, Xiangqun Nong, Zehua Zhang

**Affiliations:** 1 State Key Laboratory of Biology of Plant Diseases and Insect Pests, Institute of Plant Protection, Chinese Academy of Agricultural Sciences, Beijing, 100193 P.R. China; 2 Scientific Observation and Experimental Station of Pests in Xilin Gol Rangeland, Institute of Plant Protection, Chinese Academy of Agricultural Sciences, Xilinhot, 02600 P.R. China

## Abstract

We studied the role of plant primary and secondary metabolites in mediating plant-insect interactions by conducting a no-choice single-plant species field experiment to compare the suitability, enzyme activities, and gene expression of *Oedaleus asiaticus* grasshoppers feeding on four host and non-host plants with different chemical traits. *O. asiaticus* growth showed a positive relationship to food nutrition content and a negative relationship to secondary compounds content. Grasshopper amylase, chymotrypsin, and lipase activities were positively related to food starch, crude protein, and lipid content, respectively. Activity of cytochrome P450s, glutathione-S-transferase, and carboxylesterase were positively related to levels of secondary plant compounds. Gene expression of UDP-glucuronosyltransferase 2C1, cytochrome P450 6K1 were also positively related to secondary compounds content in the diet. Grasshoppers feeding on *Artemisia frigida*, a species with low nutrient content and a high level of secondary compounds, had reduced growth and digestive enzyme activity. They also had higher detoxification enzyme activity and gene expression compared to grasshoppers feeding on the grasses *Cleistogenes squarrosa*, *Leymus chinensis*, or *Stipa krylovii*. These results illustrated *Oedaleus asiaticus* adaptive responses to diet stress resulting from toxic chemicals, and support the hypothesis that nutritious food benefits insect growth, but plant secondary compounds are detrimental for insect growth.

## Introduction

Nearly half of all insect species are herbivores^1, 2^. Co-adaptations between herbivorous insects and host plants have been studied in depth. These studies have mainly focused on understanding behavioral, physiological, chemical, genetic, ecological, and evolutionary mechanisms^2–6^. Herbivorous insects with wide host ranges show different preferences and adaptation to different host plants^7^. Some plant species are strongly attractive or are indispensable to specific herbivorous species, and may contribute to, or even accelerate, population outbreaks^8^. For example, *Locusta migratoria manilensis* (Meyen) population growth is strongly correlated with the grass *Phragmites australis* (Cav.) Trin., which provides an optimal food source^9, 10^.

The question of which selective factors have driven the evolution of host adaptability by insect herbivores is of great interest. Ecological factors such as susceptibility to predation and aspects of habitat association are important in selection but plant chemistry is critical and includes nutrition, nutritional barriers, and secondary compounds^2, 11, 12^. Plant chemistry is an important component of the phenotype that mediates plant-insect interactions. Phenotypic and physiological plasticity of insect individuals, and genetic variability of populations, help herbivorous insects overcome plant defenses and variable diets^13^. For insect pest species, determining the causal factors and the insect response to those stresses can be helpful for explaining their population dynamics or spatial distribution, and improving management strategies^14, 15^.

Plants have evolved to resist insect feeding or deter oviposition, while insects have evolved to more effectively locate and use suitable host plants for feeding and oviposition. This ongoing evolutionary battle has lasted over 350 million years^1, 16^. To resist attack by herbivorous insects, many plants have evolved chemical defense systems, including direct and indirect responses^17^. For direct responses, plants have created nutritional hurdles, such as having low available protein or low carbohydrate content. Physical barriers include plant structures such as thorns, trichomes, and cuticles^1, 18, 19^. Plants also produce secondary metabolites including flavonoids, tannins, phenols, alkaloids, terpenoids, and glucosinolates, that function as toxins or repellents^20, 21^. Indirect defenses may involve volatile organic compounds, induced by insect feeding, and extrafloral nectaries that attract predators and parasitoids of the herbivores^2^. The “secondary compounds” produced by plants play a dual role in providing an attraction signal for insects adapted to these plants. Plant adaptations based on these two defense systems have helped plants minimise impacts and survive insect herbivory^21, 22^.

Conversely, insect herbivores have evolved novel detoxification mechanisms that allow them to consume and develop on toxic plants. This insect feeding continues the selective pressure on plants to develop increased or novel chemical defenses^23–27^. Insect herbivores have well-defined nutritional requirements for carbohydrates, lipids, proteins, vitamins, and minerals^11, 28^, and have adapted to feed on plants with varying nutritional qualities, and can accurately choose optimal food when given a choice^29, 30^.

The significant impact of plant nutrition and defensive traits on herbivore performance and population dynamics is well established. The phenotype and population dynamics of insect herbivores depend on the nutritive value and defensive traits of their host plants^2, 4, 29^. Insect survival, fecundity, fitness, and population levels usually increase when they feed on plants of optimal quality and low toxin concentrations^12, 31, 32^. The availability of such plants may increase the probability of pest population outbreaks, whereas reduced access to key nutrients or increased levels of secondary compounds may have the opposite effect^14, 28^.

Diet stress from plant chemical exposure can also effect herbivore physiology and gene expression. Gene expression and enzyme activity levels in insects can have important roles in nutrient metabolism and detoxification of secondary plant compounds^33–35^. For example, diet-dependent metabolic responses of insect herbivores such as *Spodoptera spp*., showed that the expression patterns of digestive and detoxifying enzymes, transporters, immunity, and peritrophic membrane associated transcript were varied amongst the different *Spodoptera* strains^33^. Genetic adaptation has allowed the induction of arrays of broader or more robustly active digestive and detoxifying enzymes in herbivores^36, 37^. Furthermore, the evolution of insect diet choices and the enzyme system is closely related^38^, and alterations in the expression levels of digestive and detoxifying enzymes are used by insects to optimize nutrient utilization inside the gut^39^. The expression of mannosidases, glucosidases, and alpha amylases, enzymes used to metabolize carbohydrates such as starches, are differentially induced in insect midguts by different food plants^33, 39^. Rapid synthesis of mixed-function oxidases and detoxifying enzymes occur after the consumption of toxic plants and lipid-synthesizing enzymes are generated in response to lipid-deficient diets^21, 34, 35^. These rapid biochemical and gene expression responses to changing plant chemical traits are vitally important for herbivores.

Phenotypic plasticity, physiological plasticity, and gene expression in response to diet stress are well-documented, but their specific and quantitative relations to plant chemical traits of nutrition and secondary compounds are unclear. We used *Oedaleus asiaticus* B. Bienko, a common locust of north Asian grasslands^14^, as a model species to conduct a field cage trial and study enzyme activity and gene expression. We wanted to determine how diet stress, produced by secondary plant chemicals, influences the gene expression, physiology, and phenotype of *O. asiaticus*.

## Results

### Host plant biochemical traits

**Figure 1:**
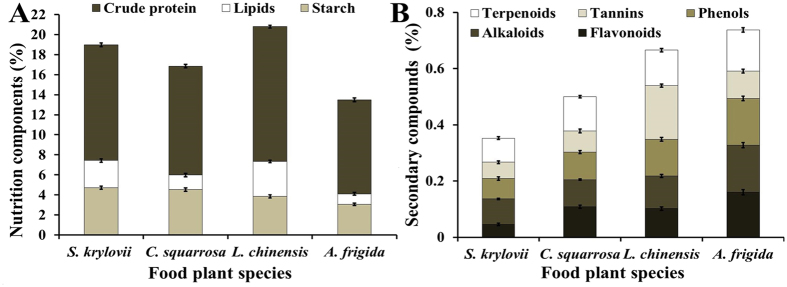
(**A**) Percentage nutrition components (crude protein, lipid, starch) and (**B**) secondary compounds (terpenoids, tannins, phenols, alkaloids, flavonoids) content (±SD, %) of the plant species *C. squarrosa*, *L. chinensis*, *S. krylovii*, and *A. frigida*, respectively.

The main chemical traits of three nutritive components and five secondary compounds for four food plants were measured by HPLC. The main nutrition and secondary compounds were different in the four plant species (Fig. 1). *S. krylovii* had the highest starch, and *L. chinensis* had the highest crude protein and lipid content (Fig. 1A). The lowest values of the three nutritive substances occurred in *A. frigida*. The sum of the three nutritive substances content was, in decreasing order, *L. chinensis* > *S. krylovii* > *C. squarrosa* > *A. frigida* (Fig. 1A).

For the five secondary compounds, the total amount of all compounds was, in decreasing order, *A. frigida* > *L. chinensis* > *C. squarrosa* > *S. krylovii* (Fig. 1B). Additionally, *A. frigida* had high levels of flavonoids, phenols, alkaloids, and terpenoids, while *L. chinensis* had the highest level of tannins compared to the other plants.

### Grasshoppers growth performance

**Figure 2:**
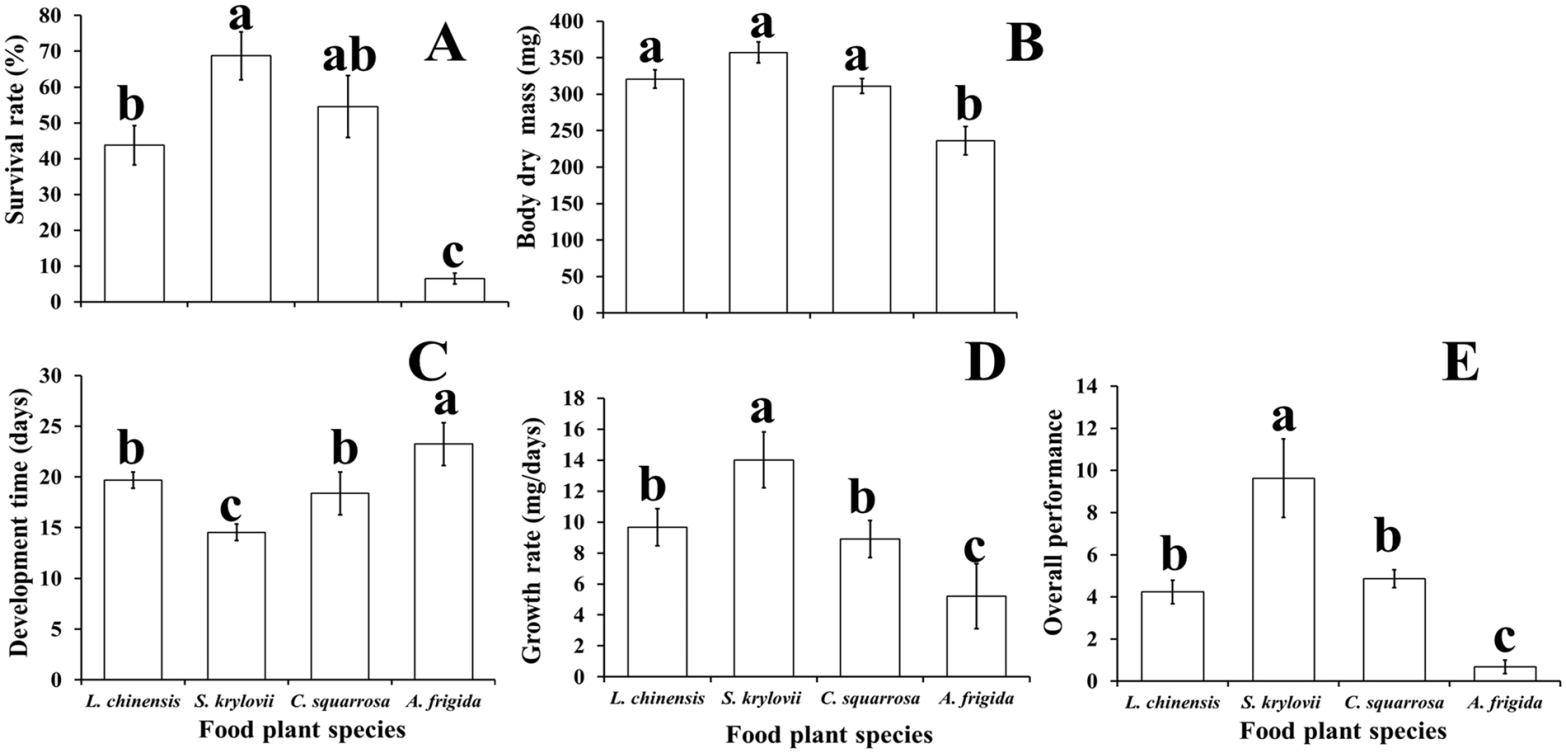
(**A**) *O. asiaticus* mean % survival rate from fourth instar to adult ± SD, (**B**) mean dry mass (mg ± SD) of adults, (**C**) mean developmental time (days ± SD) from fourth instar to adult, (**D**) growth rate (mg/day ± SD) and (**E**) overall performance (±SD) when fed on either *L. chinensis*, *S. krylovii*, *C. squarrosa*, or *A. frigida*, respectively. Bars marked by different lowercase letters are significantly different based on Turkey’s HSD analysis at *P* < 0.05.

The mean survival rate (Fig. 2A), developmental time (Fig. 2B), adult dry mass (Fig. 2C), growth rate (Fig. 2D), and overall performance (Fig. 2E) of *O. asiaticus* were significantly poorer for insects feeding on *A. frigida*, compared to *L. chinensis, S. krylovi*, or *C. squarrosa*. Feeding on *A. frigida* provided less benefit for *O. asiaticus* growth and development, presumably because of poor adaptation to this plant compared to the three grasses. Among the grasses, growth rate and overall performance were significantly higher for *O. asiaticus* feeding on *S. krylovi* (Fig. 2A–E).

**Figure 3:**
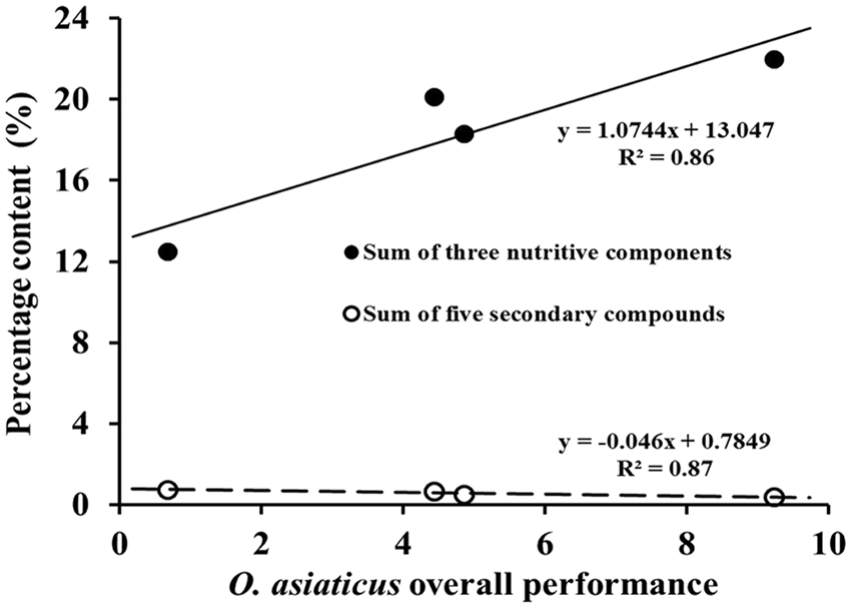
Linear relationship of *O. asiaticus* mean overall performance with the sum of three nutritive components (crude protein, lipid, starch) and five secondary compounds (alkaloids, flavonoids, phenols, tannins, terpenoids), respectively.

Linear regression analysis (Fig. 3) showed that *O. asiaticus* mean overall performance had a significant positive relationship to the sum of the three measured nutritive components of starch, crude protein and lipids (y = 1.0744x + 13.047, R² = 0.86, *P* < 0.05). In contrast, *O. asiaticus* overall performance had a significant negative relationship to the sum of five secondary compounds of flavanoids, tannins, phenols, alkaloids and terpenoids (y = −0.046x + 0.7849, R² = 0.87, *P* < 0.05).

### Grasshopper enzyme activity

**Figure 4:**
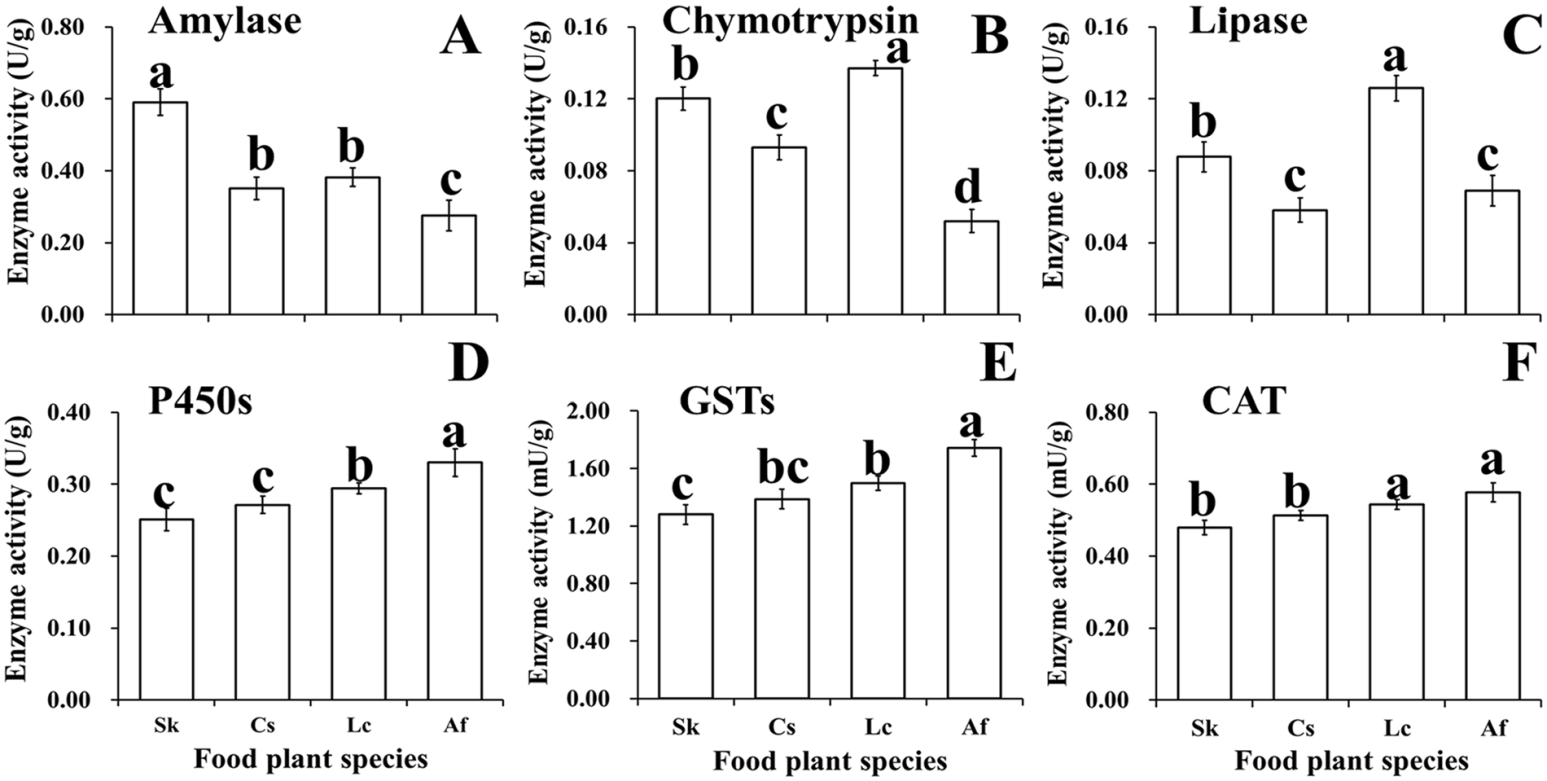
The activity (±SD) of the main digestive (amylase, chymotrypsin, lipase) and detoxification (P450s: cytochrome P450s, GSTs: glutathione-S-transferase, CAT: carboxylesterase) enzymes for *O. asiaticus* when fed on *L. chinensis* (Lc), *S. krylovii* (Sk), *C. squarrosa* (Cs), and *A. frigida* (Af). Bars marked by different lowercase letters are significantly different based on Turkey’s HSD analysis at *P* < 0.05.

*O. asiaticus* fed on *S. krylovii* had the highest amylase activity (Fig. 4A), while *O. asiaticus* fed on *L. chinensis* resulted in the highest chymotrypsin (Fig. 4B) and lipase activity (Fig. 4C). Individuals fed *A. frigida* had the lowest activities of these three digestive enzymes (Fig. 4A–C). The detoxification activities of P450s, CAT, and GSTs were highest in *O. asiaticus* fed on *A. frigida* (Fig. 4D–F), followed by *O. asiaticus* fed *L. chinensis* with significant differences observed in P450s and GSTs enzymes. The lowest values were for *S. krylovii* (Fig. 4D–F).

**Figure 5:**
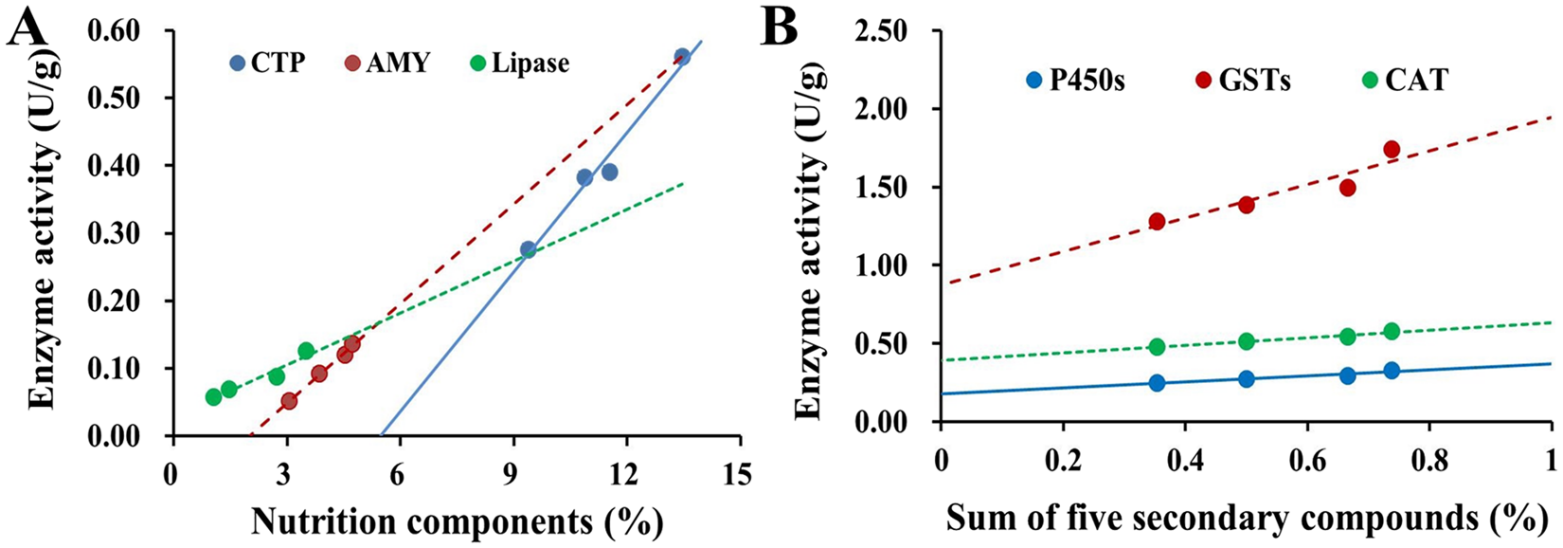
Linear relations between *O. asiaticus* mean enzyme activity and food plant chemical traits. (**A**) Relationship between mean chymotrypsin (CTP, U/g) activity and mean crude protein content, mean amylase (AMY, U/g) activity and mean starch content, mean lipase activity (U/g) and mean lipid content. (**B**) Relationship between cytochrome P450s (P450s, U/g), glutathione-S-transferase (GSTs, mU/g), carboxylesterase (CAT, mU/g) mean activities with the sum of five secondary compounds (%).

Linear regression analysis (Fig. 5) showed that the activities of amylase (AMY), chymotrypsin (CTP) and lipase had a significant positive relationship to starch (y = 0.0049x − 0.0972, R² = 0.99), crude protein (y = 0.0688x − 0.3755, R² = 0.97), and lipids (y = 0.0255x + 0.0295, R² = 0.93), respectively (*P* < 0.05) (Fig. 5A). In contrast, the activities of P450s (y = 0.1899x + 0.1796, R² = 0.92), GSTs (y = 1.0711x + 0.8731, R² = 0.87), and CAT (y = 0.2396x + 0.3932, R² = 0.97) were all significantly positively related to the sum of the five secondary compounds (*P* < 0.05) (Fig. 5B).

### Grasshopper gene expression

**Figure 6:**
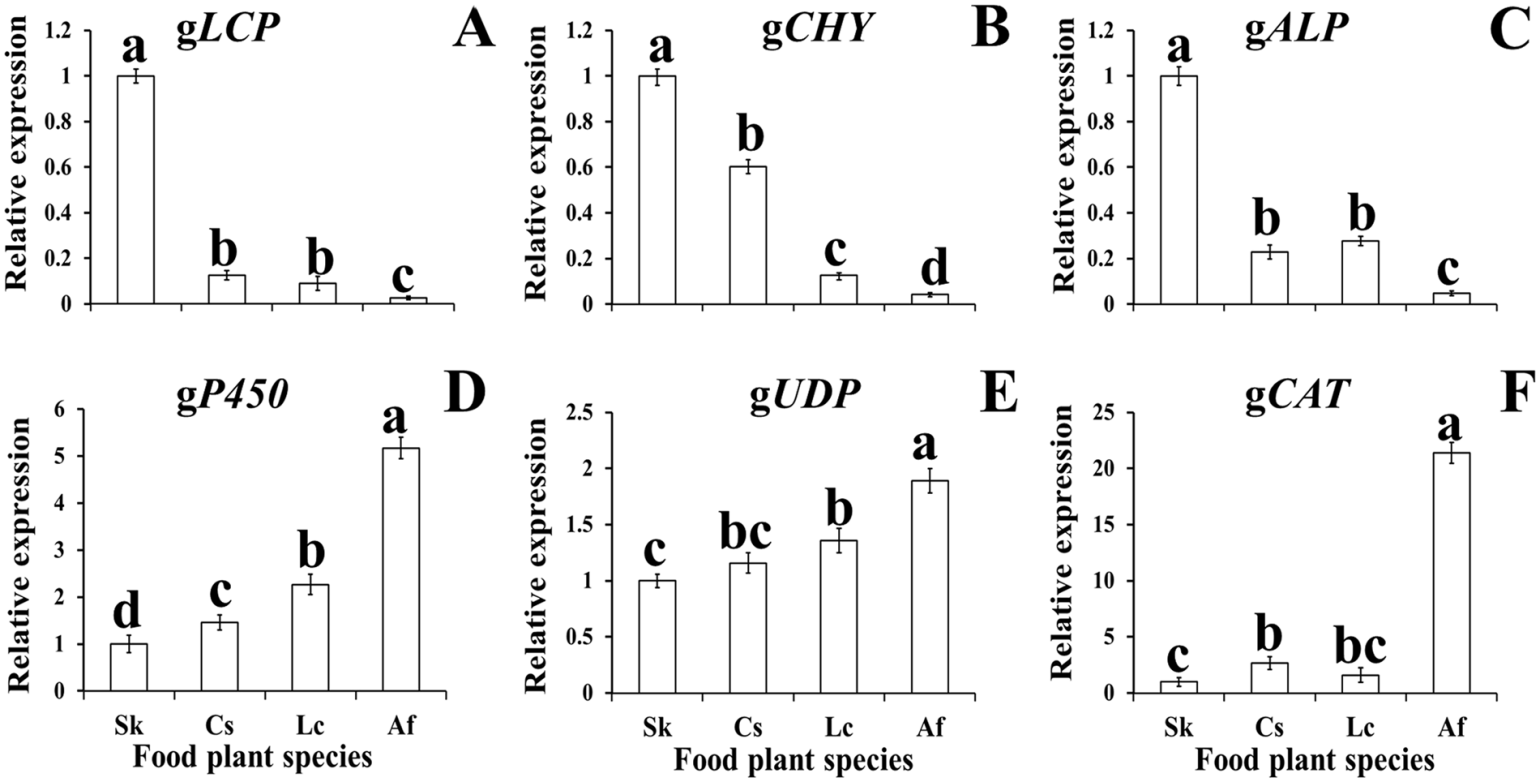
Relative expression (±SD) of six candidate genes for *O. asiaticus* feeding on *S. krylovii* (Sk), *L. chinensis* (Lc), *C. squarrosa* (Cs), and *A. frigida* (Af). Different lower case letters indicate a significant difference among the four treatments at P = 0.05. Key: g*LCP*: Cuticle protein 6; g*CHY*: TPA_exp: chymotrypsin 2; g*ALP*: alpha-glucosidase; g*UDP*: UDP-glucuronosyltransferase 2C1; g*P450*: cytochrome P450 6K1; g*CAT*: carboxylesterase.

RT-qPCR to determine the relative expression of six genes indicated that the genes for cuticle protein 6 (g*LCP*), TPA_exp: chymotrypsin 2 (g*CHY*), and alpha-glucosidase (g*ALP*) were most highly expressed in *O. asiaticus* fed on *S. krylovii* (Fig. 6A–C). The lowest expression levels for these proteins were for *O. asiaticus* fed on *A. frigida*. In contrast, the genes of UDP-glucuronosyltransferase 2C1(g*UDP*), cytochrome P450 6K1 (g*P450*), and CAT (*gCAT*) were highest in *O. asiaticus* fed *A. frigida*, with the lowest expression in *O. asiaticus* fed *S. krylovii* (Fig. 6D–F).

Linear regression analysis showed that the mean relative gene expression of g*p450* (y = 2.0309x + 0.2062, R² = 0.81) and g*UDP* (y = 9.8045x − 2.932, R² = 0.82) exhibited a significant positive relationship to the content sum of secondary compounds in the food plant (*P* < 0.05). Conversely, the relationship between carboxylesterase (gCAT) and the % sum of secondary compounds was not significant.

## Discussion

We used single-plant no-choice field cage trials to compare the suitability of four plant species with different chemistry to *O. asiaticus*. The results demonstrate that *A. frigida*, with low nutritive value and a high level of secondary compounds, was unsuitable for *O. asiaticus* compared to the grasses (*L. chinensis*, *S. karylovii*, and *C. squarrosa*). Grasshoppers that fed only on *A. frigida* had reduced size, growth, development, and survival compared to those fed on any of the grass species. Among the grasses, grasshoppers fed on *S. karylovii* had increased growth and development compared to the other two grass species. From the viewpoint of grasshopper biology, *S. karylovii* was the most favorable host plant of all plants tested. These results were consistent with previous studies^40, 41^. *S. krylovii* is an iconic grass species found in the Eurasian steppe grassland, with a wide geographic distribution that includes northern China, Kazakhstan, Mongolia, and Siberia^42, 43^. Due to the close relationship between *S. krylovii* and *O. asiaticus*, we inferred that *S. krylovii* was a strong driver for the distribution of *O. asiaticus*. Those areas of *Stipa*-dominated grasslands, represent regions for potential expansion of *O. asiaticus* range, particularly in the context of climate change, which may promote outbreak populations^44^. Thus, *O. asiaticus* monitoring and population management should be increased in these regions.

*O. asiaticus* growth performance was positively related to total nutrition of the food plant, and negatively related to the total amount of secondary compounds. These results follow a tenet of herbivore-plant coevolution in that better nutritional status benefits insect growth whereas a higher level of secondary compounds is generally detrimental^11, 21, 22^. In addition, the individuals of grass- and *A. frigida*-fed grasshoppers have significantly different phenotypes, could be viewed in the context of insect phenotypic plasticity. Phenotypic plasticity refers to the capacity of a single genotype to change their phenotypes (ie. biochemistry, metabolism, physiology, morphology, development, behavior, or life-history) in response to harmful environmental stresses or conditions, which would be highly beneficial for insects^13, 45^. It has the great advantage of allowing continuous, on-going adaptation of individuals in real-time. Indeed, some phenotypic plasticity is instantaneous, and many types are reversible^13^. However, we do not know if phenotypic changes observed in this study become fixed in the next generation, or disappear when *O. asiaticus* is transfered to suitable host grasses.

There was a significant positive relationship between the digestive enzyme (chymotrypsin, amylase, and lipase) activity and food plant nutrition components (crude protein, starch and lipids). In contrast, *O. asiaticus* detoxification enzyme (CAT, GSTs, and P450s) activity showed a significant positive relationship to total secondary compounds content. This indicated that the low nutrition and high secondary compound levels associated with *A. frigida* resulted in low activity of nutrition digestive enzymes and high activity in detoxification enzymes. In contrast, the high nutrition and low secondary compounds content of *S. krylovii* resulted in high nutrition digestive enzymes activity and low detoxification enzymes activity. These results support the general viewpoint that the evolution of food adaptability is tightly correlated with nutrition metabolism and detoxification related enzymes^33, 34, 36, 38^. For example, enhanced survival and fecundity of *Hyposidra infixaria* (Lepidoptera: Geometridae), when larvae were reared on an artificial diet compared to tea leaves, was linked to higher activity of enzymes related to nutrition metabolism^46^. In addition, the expression of cytochrome P450, GST and CAT are differentially induced by feeding on the different host plants of herbivorous insects^21^. When feeding on plants having low suitability, the expression of detoxification-related enzymes, such as cytochrome P450, and GSTs, may be activated in response to the presence of toxic substances.

Altered gene expression offers additional insights into the biological performance of *O. asiaticus*. As previously mentioned, insects often up-regulate detoxifying enzymes, such as cytochrome P450, in response to toxin exposure. UDP-glucuronosyltransferase (UDP) is known to be highly inducible and it functions to ameliorate stress^21^. Up-regulation of both of these genes is clearly beneficial for the grasshoppers, and probably represents adaptive phenotypic plasticity. The relative gene expression of UDP-glucuronosyltransferase 2C1 (*gUDP*) and cytochrome P450 6K1 (g*P450*) exhibited a significant positive relationship to secondary compounds in the host plant. Feeding on *A. frigida* significantly up-regulated stress-resistance genes regulating UDP, P450s, and CAT. Up-regulating these genes as a result of feeding on *A. frigida* is unsurprising, given that this plant is not a preferred host^40, 41^.

*O. asiaticus* feeding on *A. frigida* significantly down-regulated the gene expression of cuticle protein 6 (g*LCP*), TPA_exp: chymotrypsin 2 (g*CHY*), and alpha-glucosidase (g*ALP*).Those partly represented the decreasing of structural constituent of cuticle pathway, protein digestion and the absorption pathway, and starch and sucrose metabolism pathway, can explain the poorer performance. But, we do not know if the down-regulation of g*LCP*, g*CHY*, and g*ALP* is beneficial, detrimental, adaptive, or simply an incidental by-product of diet stress, but may be related to variation in plant nutrition or secondary compounds. However, even seemingly harmful consequences that result from altered genes, may be beneficial. An example is a gene change that delays growth, development, or reproduction. This response may at first appear to be detrimental to the organism, but it may be beneficial if it allows the individual to survive during a period of stress, such as during poisoning or poor nutrition. Conversely, it may make the insect more susceptible to predation, parasitism or unable to complete life history before the onset of unfavourable environmental conditions^47^.

We offer several conclusions based on this study. First, different food plants can elicit biochemical responses consistent with phenotype plasticity and confirms previous studies linking a changed phenotype to changed environment^33^. Second, feeding on suboptimal plant species and the associated diet stress substantially altered gene expression (i.e. grasshoppers fed *A. frigida* compared with grass-fed insects), which aligns with conclusions of previous studies showing gene expression changes with increased diet stress^48^. Third, the quality of nutrition and levels of toxin in host plants can significantly affect insect growth, physiological enzyme activity, and gene expression. The roles of plant primary and secondary metabolites in mediating plant-insect interactions hence have both ecological and evolutionary consequences^42^. To decipher the mechanisms of herbivorous insect food adaptability at the cellular level, we must determine exactly how the terpenoids, flavonoids, and alkaloids present in *A. frigida* influence physiology and biology as well as regulate insect gene expression in *O. asiaticus*.

## Materials and Methods

### Ethics statement

*Oedaleus asiaticus* were collected at the Xilin Gol grassland in 2016. Species of the superfamily Acridoidea are common agricultural pests and are not on the “List of Protected Animals in China”. No permits were required for the described field studies.

### Study sites

The research site (43.968°N, 115.821°E) was located in the Xilin Gol League, Inner Mongolia, northeast China. This region is representative of the Eurasian steppe grassland^14^. The mean annual temperature in the study area is 0.3 °C with mean monthly temperatures ranging from −21.6 °C in January to 19.0 °C in July. The mean annual precipitation is 346 mm, more than 80% of that which occurs during the May to September growing season^15^. Vegetation at the study site is mainly comprised of three grass species *Cleistogenes squarrosa* (Trin.) Keng, *Leymus chinensis* (Trin.) Tzvel, and *Stipa krylovii* Roshev (all Poaceae), as well as *Artemisia frigida* Willd (Compositae). The three common grasshoppers species are *O. asiaticus*, *Calliptamus abbreviatus* Ikonn., and *Dasyhippus* barbipes (Fischer-Waldheim). All three species overwinter as eggs with egg hatch occuring between late-May and late-June, with third instars leading to adults appearing in July^49^. As a major grasshopper pest, *O. asiaticus* populations have sometimes reached outbreak densities, producing devastating impacts on grassland ecosystems^14, 15^.

### Field cage study of *O. asiaticus* growth performance in grassland

During late June, 2016, we studied *O. asiaticus* growth when nymphs were reared on different host plant species. A field cage study was carried out on *S. krylovii*, *A. frigida*, *C. squarrosa*, and *L. chinensis* grasslands. In each of those four grasslands, we removed all other plants to assure that only one host plant remained. Near the end of June, a total of 20 screen cages (1 m × 1 m × 1 m) were constructed using iron rod frames covered with 1 mm^2^ cloth mesh. Five cages were used per plant species. We removed all visible spiders and other natural enemies from the field cages before adding female fourth instar *O. asiaticus*. The mesh covering the cages reduced both wind flow and sunlight intensity across all cages equally. Therefore, impacts of these factors on plant growth among the cages and treatments were considered minimal, as was found in previous studies^14^.

Fourth instar *O. asiaticus* nymphs were collected by sweep net from the grassland containing these four host plants on 20 June, 2016. The grasshopper nymphs developed very uniformly in this grassland. Collected individuals were then temporarily maintained in metal-frame cages (40 × 40 × 40 cm), covered with fine fabric mesh. They were fed fresh-cut vegetation daily, consisting of 90 g *S. krylovii*, 90 g *L. chinensis*, 90 g *C. squarrosa* and 90 g *A. frigida*. Cages were placed in a shadehouse for 2 d, until the nymphs were transferred to the experiment (see below).

Fourth instar *O. asiaticus* nymphs (starved for 24 h) randomly assigned to the 20 cages (16 individuals per cage). Those experimental individuals were selected to be as uniform in size as possible, with fresh body mass weighed and then verified by ANOVA to confirm there were no significant differences in the weight of *O. asiaticus* nymphs amongst the four treatments. Because the gender of the early instars is difficult to identify, fourth instar females were selected based on external morphology of the reproductive system. Before the start of the field cage study, a cohort of 30 *O. asiaticus* fourth instar females were euthanized by chloroform and dried at 90 °C for 24 h, after which they were individually weighed (mg), and a mean dry mass determined to serve as the baseline data for calculating the increased body dry mass from fourth instar to adult. Once grasshoppers were assigned to treatments, we inspected field cages daily to monitor survival and remove dead individuals. Once all surviving individuals became adults, they were also euthanized by chloroform and dried at 90 °C for 24 h to determine adult dry mass (mg). The body mass increase (mg) was calculated by subtracting the fourth instar body dry mass from the adult body dry mass. Survival rate (%) from fourth instar to adult was calculated by the number of individuals surviving through to adulthood / number of initial fourth instar individuals (n = 16). Development time (days) was calculated by the following formula^50^:

$$DT=\frac{\sum _{i=1}^{n}i\ast {N}_{i}}{{N}_{t}},$$

where *i* is the number of days from fourth instar to adult; *N*_i_ is the number of individuals with the development time corresponding to that value of “*i*”; and *N*_t_ is the number of all grasshoppers surviving to adulthood. Growth rate (mg/day) was calculated by body mass increase/development time, and overall performance calculated from growth rate × survival rate^14^.

### Host plant biochemical traits

For each treatment, the nymphs were able to feed ad libitum on the plant biomass of the grassland, with the vegetation in the cages providing sufficient vegetation to allow development through to adults. Once the adults were removed, the remaining plants from each cage were cut at ground level, and each species placed in a separate plastic container and returned to the laboratory for chemical analysis. Starch, nitrogen, and lipid content of each plant sample were measured using the Iodine-starch colorimetric method, Kjeldahl method, and Soxhlet extraction method^51, 52^, respectively. Crude protein content were then calculated as nitrogen content × 6.25^53^. Secondary compounds specifically the flavonoids, tannins, phenols, alkaloids, and terpenoids of each sample were measured by high performance liquid chromatography (HPLC), using the techniques of Ossipov *et al*.^54–58^.

### Grasshopper enzyme activity

We analyzed the main digestive enzyme activity of amylase, chymotrypsin, and lipase, and the main detoxifying enzyme activity of cytochrome P450s (P450s), glutathione-S-transferase (GSTs), and carboxylesterase (CAT). One adult was randomly collected from each of the five replicates of the four treatments (20 samples). Each sample was homogenized separately in fresh 0.1 M sodium phosphate buffer, then centrifuged at 10,000 g for 15 min at 4 °C. The supernatant was stored at −20 °C for future use. Protein present in the enzyme suspension was measured using the method by Lowry *et al*.^59^. Amylase, chymotrypsin, and lipase activity was assayed using methods described by Prasad *et al*.^46^, and calculated as units of U/g.

GSTs activity was measured using a modification of the method described by Oppenoorth and Welling^60^. After pipetting 100 μL of 1-chloro-2,4-dinitrobenzene (CDNB) (20 mM) or 3,4-dichloronitrobenzene (DCNB) (40 mM), and 100 μL of GSH (40 mM) into microplate wells, we added 50 μL of enzyme solution (for DCNB) or 10 μL of enzyme solution and 90 μL of PBS (for CDNB). The OD values at 340 nm were recorded at 25 s intervals for 10 min. The P450s and CAT activities were determined using commercial assay kits (Nanjing Jiancheng, Nanjing, China) according to manufacturer instructions. The P450, GST, and CAT enzyme activities were calculated in units of U/g, mU/g, and mU/g, respectively.

### Gene expression study

We investigated six genes of *O. asiaticus* to compare their relative expressions when exposed to diet stress from different chemical traits, including the cuticle protein 6 (*gLCP*) that is a structural constituent of cuticle pathway, TPA_exp: chymotrypsin 2 (*gCHY*) involved in protein digestion and the absorption pathway, alpha-glucosidase (*gALP*) involved in the starch and sucrose metabolism pathway, and the UDP- glucuronosyltransferase 2C1 (*gUDP*), cytochrome P450 6K1 (*gP450*), and carboxylesterase (*gCAT*) involved in the xenobiotics metabolism pathway. Their unigene sequences were acquired from our previous transcriptome profiles (RSA accession number SRP072969) and chosen to design gene-specific primers (Table S1).

The relative expression of the six candidate genes were analyzed by qRT-PCR. We collected one adult sample randomly from each replicate of the 4 treatments (20 samples). Total RNA was extracted from each sample, using TRIzol reagent (Invitrogen, California, USA) following manufacturer instructions. The cDNA was synthesized using AMV reverse transcriptase (Invitrogen, Carlsbad, CA, USA). Gene-specific primers of the 6 genes were designed using Primer Express Software v2.0 (Applied Biosystems, Foster City, CA, USA). All primers used are listed in Table S1. Experiments were performed in the StepOne Plus Real-Time PCR system (Applied Biosystems) using SYBR green PCR mix (QIAGEN, Hilden, Germany). Then, b-actin was amplified for internal standardization. PCR efficiency and specificity of primers of the target genes were validated in the experiment. The qRT-PCR was performed in a 25 μl reaction mixture, and PCR was conducted under the following conditions: denaturation at 95 °C for 2 min, followed by 40 cycles of 94 °C for 10 s, annealing at 59 °C for 10 s (each primer has itself annealing temperature), and extension at 72 °C for 40 s. At the end of each reaction, the melting curve was analyzed to confirm the specificity of the primers. Relative gene expressions were normalized by the internal standard of actin, and analyzed using the 2^−ΔΔCT^ Method. Expression values were adjusted by setting the expression of *O. asiaticus* feeding on *S. krylo*vii to be 1 for each gene. All RT-qPCRs for each gene of 20 samples (five biological replicates for each treatment) used 3 technical replicates per experiment.

### Data analysis

We used one-way analysis of variance (ANOVA) and Turkey’s HSD to compare grasshopper growth variables (body size, survival rate, development time, growth rate, and overall performance), enzyme activity, and relative gene expression when *O. asiaticus* was confronted with diet stress of different chemicals. Correlation analyses of grasshopper overall performance, enzyme activity, and relative gene expression with food plant nutrition or secondary compounds were also conducted. We used SAS version 8.0 for all analyses.

## Electronic supplementary material

Supplementary Information
