# Supplementary material for: Biology, physiology and gene expression of grasshopper Oedaleus asiaticus exposed to diet stress from plant secondary compounds
Source: Sci Rep. 2017 Aug 17;7:8655. doi: 10.1038/s41598-017-09277-z (PMC5561062; doi:10.1038/s41598-017-09277-z)
Supplement: Supplementary file 1 — Supplementary Information [file 41598_2017_9277_MOESM1_ESM.pdf]

Biology, physiology and gene expression of grasshopper *Oedaleus asiaticus* exposed to diet stress from plant secondary compounds

Xunbing Huang, Jingchuan Ma, Xinghu Qin, Xiongbing Tu, Guangchun Cao, Guangjun Wang, Xiangqun Nong & Zehua Zhang

Table S1 Designed sequences of qRT-PCR primers for the six genes

Grass species	Sequence of primers for real-time PCR (5' to 3')	
Cuticle protein 6	Forward	AGTGTACTGAGCGCTCTGG
g*LCP*	Reverse	GTCTCCATCTCTTCGCAGT
TPA_exp: chymotrypsin 2	Forward	ACGCCAACATCGCCAACTA
gCHY	Reverse	ACGGTGTCGCTGTTGTAGGA
alpha-glucosidase	Forward	CCATCATTCAGCAGTTCCG
gALP	Reverse	CAATCACCCAGTTGGACCAC
UDP- glucuronosyltransferase	Forward	TGATACCACCGCCACATTC
2C1 *gUDP*	Reverse	AACCCACTGAGGTCCGTCAT
cytochrome P450 6K1	Forward	CCTTCTTGCTGGCTATGAAAC
gP450	Reverse	CCACATCAATGGTCACTTCTG
Carboxylesterase	Forward	ACAGTGGCATCTCGGATG
gCAT	Reverse	TCAGTCGGCGATGTGGAG
